# PKG and PKC Are Down-Regulated during Cardiomyocyte Differentiation from Embryonic Stem Cells: Manipulation of These Pathways Enhances Cardiomyocyte Production

**DOI:** 10.4061/2010/701212

**Published:** 2010-04-26

**Authors:** Stephen Mobley, Jessica M. Shookhof, Kara Foshay, Michelle Park, G. Ian Gallicano

**Affiliations:** ^1^Department of Biochemistry and Molecular & Cellular Biology, Georgetown University Medical Center, Med/Dent Building NE205, 3900 Reservoir Road NW, Washington, DC 20057-1455, USA; ^2^Biotechnology and Life Sciences Laboratory, Science and Technology Division, Thomas Jefferson High School for Science and Technology, 6560 Braddock Road, Alexandria, VA 22312, USA

## Abstract

Understanding signal transduction mechanisms that drive differentiation of adult or embryonic stem cells (ESCs) is imperative if they are to be used to cure disease. While the list of signaling pathways regulating stem cell differentiation is growing, it is far from complete. Indentifying regulatory mechanisms and timecourse commitment to cell lineages is needed for generating pure populations terminally differentiated cell types, and in ESCs, suppression of teratoma formation. To this end, we investigated specific signaling mechanisms involved in cardiomyogenesis, followed by manipulation of these pathways to enhance differentiation of ESCs into cardiomyocytes. Subjecting nascent ESC-derived cardiomyocytes to a proteomics assay, we found that cardiomyogenesis is influenced by up- and down-regulation of a number of kinases, one of which, cGMP-dependent protein kinase (PKG), is markedly down-regulated during differentiation. Delving further, we found that manipulating the PKG pathway using PKG-specific inhibitors produced significantly more cardiomyocytes from ESCs when compared to ESCs left to differentiate without inhibitors. In addition, we found combinatorial effects when culturing ESCs in inhibitors to PKG and PKC isotypes. Consequently, we have generated a novel hypothesis: Down-regulation of PKG and specific PKC pathways are necessary for cardiomyogenesis, and when manipulated, these pathways produce significantly more cardiomyocytes than untreated ESCs.

## 1. Introduction

To achieve the goals of stem cell therapies, the varying mechanisms that regulate the transition of adult or embryonic stem cells from undifferentiated to their differentiated states must be understood. This concept is important for producing bona fide terminally differentiated cell types from ES cells preventing teratoma formation. A prime candidate for stem cell therapy is to repair infarcted areas as a result of heart disease. The challenge has been to find repair mechanisms that prevent cell death inherent in cardiac remodeling and introduce nascent cardiomyocytes that restore heart function. A possible answer to this challenge is adult or ES cell-derived cardiomyoplasty [1–4]. However, without detailed comprehension of mechanisms that regulate ES cell differentiation, quality control issues abound once they are considered for clinical use. 

As in the embryo, ES cell differentiation is thought to be an inductive process, in which development of each germ layer influences the other germ layers. Once we understand how a stem cell commits to a certain fate, forever surrendering its pluripotency, we can use this knowledge to enhance fate-directed ES-like cell differentiation. 

With this concept in mind, we previously demonstrated the functional importance of the JAK/STAT3 [5] and PKC pathways in ES cells as they differentiate into beating cardiomyocytes [6]. Here, we not only reveal the functional importance of PKG, but also we show how this pathway can be manipulated to induce ES cells to form significantly more cardiomyocytes than in controls. 

PKG is a well-studied serine/threonine protein kinase in many systems [7–9]. In vascular smooth muscle, PKG1 has been shown to activate myosin light-chain (MLC) phosphatase by phosphorylating its myosin-binding subunit, consequently inhibiting MLC phosphorylation and contraction [10]. Recent evidence has shown that PKG activation can interfere with cardiac function by phosphorylating and inhibiting the cardiac L-type CA^2+^ channel current (*I*_Ca,L_) and, along with PKA, reducing the voltage-dependent fast Na^+^ current in mammalian ventricular myocytes [11]. 

**Figure 1:**
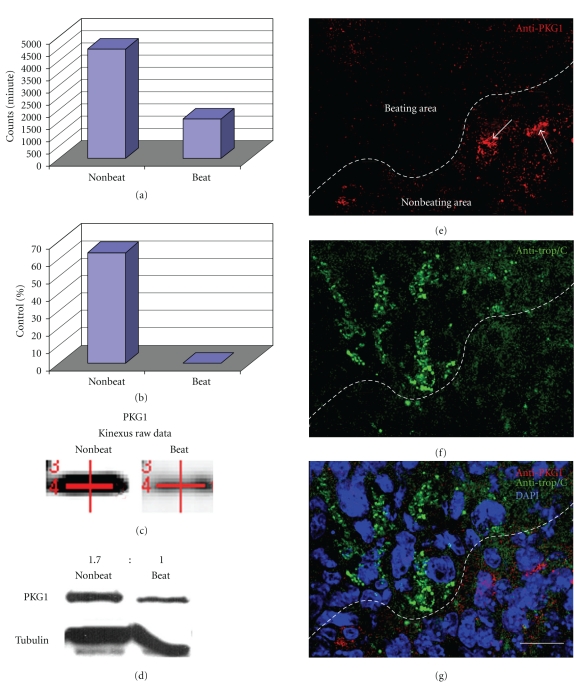
Graphs show raw and normalized data taken directly from the Kinexus protein kinase screen. (a) Kinexus Inc. (http://www.kinexus.ca/) uses a highly sensitive imaging system with a 16-bit camera (Bio-Rad Fluor-S Max Multi-Imager) in combination with quantitation software (Bio-Rad Quantity One) to quantify and analyze the chemiluminescent samples. The resulting trace quantity for each band scanned at the maximum scan time is termed the raw data. As the relationship between scan time and band intensity is linear over the quantifiable range of the signal intensity, the raw data from the scans are normalized to 60 seconds (counts per minute—C.P.M.) for uniformity. (b) After normalization, data are converted to percentages by subtracting the control (Nonbeating) normalized C.P.M. from the experimental (Beating) normalized C.P.M., followed by dividing the difference by the control (Nonbeating) normalized C.P.M. and multiplying by 100. (c) The actual PKG1 bands from the Kinexus report show a stronger signal in beating areas when compared to nonbeating areas. The red lines in (c) are generated and used by the computer to find the correct bands. (d) Followup Western blots confirmed the Kinexus data. A ratio of PKG1 protein levels was calculated by normalizing to tubulin showing that there is 1.7X (69%) more PKG1 in nonbeating areas versus beating areas. (e)–(g) Confocal microscopy of EBs showed higher levels of PKG1 in nonbeating areas, represented by anticardiac troponin (e)–(f), when compared to nonbeating areas. Arrows point to PKG1 within cells negative for cardiac troponin. Scale bar in *G* =100 micrometer.

Based on evidence linking PKG1 with attenuated muscle contraction, combined with a kinase expression screen (Figure 1) implicating the loss of PKG1 in cardiomyocytes, we hypothesized that PKG1 must be downregulated so that ES cells can complete differentiation into beating cardiomyocytes. Moreover, based on our previous work, we hypothesized that manipulating the PKG and PKC pathways would produce significantly more cardiomyocytes than untreated ES cells. To test our hypotheses, we exposed ES cells to specific PKG inhibitors and analyzed ES cell differentiation. We found that inhibiting PKG1 in ES cells during differentiation promoted cardiomyogenesis compared to controls. Furthermore combining antagonists to PKC isotypes [6] and PKG1 clearly increased production of cardiomyocytes. 

## 2. Materials and Methods

### 2.1. Cells

Pluripotent CCE-type mouse ES cells [12] were maintained as described in [5, 6, 13] and induced to differentiated following established protocols [5, 6, 13]. After 7 days in suspension, EBs were manually removed from suspension dishes and placed into each well of a 24-well plate in LIF-deficient medium. Rhythmic beating of EBs was monitored during days 11 through 18 using phase microscopy. EBs were plated, with the following experimental model: control EBs, control EBs with DMSO, EBs with PKG1 inhibitor, as well as EBs with PKG and either PKC*β* or PKC*ζ* inhibitors.

### 2.2. Reagents

The PKG1*α* cell-permeable inhibitor DT-3 was purchased from Calbiochem (San Diego, CA) and was dissolved in DMSO at 100X. DT-3 is a molecular inhibitor rather than a pharmacological agent, and is part of the regulatory subunit that specifically binds to and inhibits only PKG1*α*. The working concentration of DT-3 was 50 *μ*M. Myr-PKC pseudosubstrates were purchased from Biomol (Plymouth Meeting, PA). Each inhibitor was dissolved in DMSO at 100X immediately prior to use. Unused inhibitors were aliquoted and stored at −80°C. PKC inhibitors are amino acid sequences that bind to their respective RACKs (receptor for activated protein kinase C) [6, 14, 15]. 

### 2.3. RT-PCR, *q*RT-PCR, Western Blot Analyses, Confocal Microscopy, and Video Microscopy

Analyses were performed as described in [5, 6, 13]. For *q*RT-PCR, all RNA samples were prepared by extraction with the mirVana miRNA isolation kit (Ambion, Austin, TX). Samples were treated with DNAseI to digest and contaminate genomic DNA and diluted to 10 ng/*μ*L. All primers were designed with the aid of using the Promega Biomath website http://www.promega.com/biomath/calc11.htm. cDNA was made using the RT² First Strand Kit (C-03) and the RT^2^ SYBR Green qPCR Master Mixes (PA-012) from SA Biosciences (Frederick, MD). No-RT controls were performed for all qRT-PCR reactions. Individual samples were run in triplicate, and each experiment was repeated at least 3 times. All samples were run on the Applied Biosystems ABI Prism 7500 system using SDS 2.1 software according to the manufacturer's instructions. Samples were analyzed using the downloadable forms available on the SA Biosciences website http://www.sabiosciences.com/pcrarraydataanalysis.php. GAPDH was used as the endogenous RNA control, normalizing each sample to its expression. All samples were run on the Applied Biosystems ABI Prism 7500 system using SDS 2.1 software. Relative gene expression was calculated using the 2_T_^−ΔΔC^ method.

### 2.4. FACS Analysis

Analysis of flow cytometry data was identical to that in [16]. Members of the Georgetown University Flow Cytometry Core Facility acquired and analyzed all flow cytometry data. Briefly, EBs were trypsinized, fixed in 4% formaldehyde, and permeabilized with 0.1% Triton-X-100 in blocking buffer. Primary antibodies for cardiac troponin were from Santa Cruz Inc. and secondary antibodies conjugated to Alexa Fluor 488 fluorochrome from Molecular Probes (Eugene, Oregon). Primary antibodies were added to samples for 1 hour, followed by 5 washes in blocking buffer. Samples were then subjected to secondary antibody for 1 hour followed by 5 washes in PBS. The *x *axis is the fluorescence level of cardiac troponin. The *y *axis is the cell count. M1 marks cardiac positive cells.

### 2.5. Statistics

 All experimental groups in this study were replicated a minimum of 3 times. For display of data, each point on a graph represents a mean ± standard error of the mean (SEM; represented by error bars) for an experimental group or observation. To determine statistical differences between experimental groups or observation points, we applied the Anova statistical software package provided by Microsoft Excel (modified *t*-test). Graphs and tables showing percentages were analyzed in two ways: by pooling all data together for a given treatment group or by obtaining a percentage for each experiment and producing an SEM for all experiments.

## 3. Results

### 3.1. PKG1 Protein and mRNA Levels Are Reduced in Beating Areas

**Figure 2:**
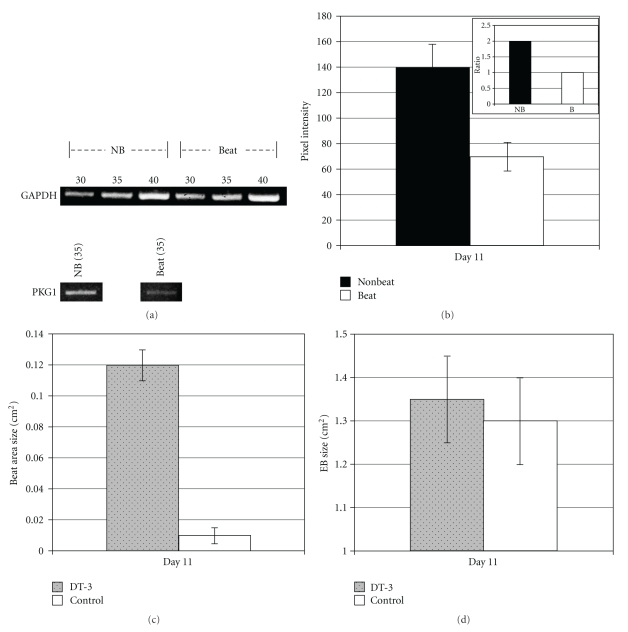
Gene expression was measured by semiquantitative RT-PCR using PKG1-specific primers. (a) Beating and nonbeating areas of similar sizes were dissected apart as described in [4, 10], followed by RNA isolation and quantification. Control GAPDH RT-PCR amplification revealed that 35 cycles were in the linear range and nucleotides in the PCR reaction were not exhausted. (b) After normalization to GAPDH levels, PKG1 mRNA levels were lower by approximately one-half in beating areas when compared to nonbeating areas (pixel intensity = 70 ± 11.0 to 139 ± 19.1, resp.). A representative ratio of PKG mRNA signal revealed 1.98X more in nonbeating areas when compared to beating areas (inset in (b)). (c) Inhibiting PKG1 resulted in dramatic and significant (*P* < .005) increase in the size of beating areas within EBs; however, the total size of EBs was not changed by PKG inhibition (d).

The raw data from the kinase expression screen indicated a dramatic decrease of PKG1 in beating areas when compared to nonbeating control areas (Figures 1(a)–1(c)). Western blot analyses by two independent laboratories verified PKG1 downregulation in cardiomyocytes (Figure 1(d)). After normalizing intensity of protein levels to tubulin, nonbeating areas were determined to have 1.7X (69%) more PKG1 protein than beating areas. Confocal microscopy corroborated the proteomics screen (Figures 1(e)–1(g)). Newly differentiated, beating cardiomyocytes identified by cardiac troponin T-(Tnnt2-) positive staining (Figure 1(f)) showed lower levels of PKG1 in beating areas when compared to Tnnt2-negative, nonbeating areas (Figures 1(e)–1(g)). Semi-quantitative RT-PCR revealed PKG1 mRNA levels lower by approximately one-half in beating areas when compared to nonbeating areas confirming Western blot analyses (Figures 2(a) and 2(b)).

### 3.2. PKG1 Inhibition Affects Cardiomyocyte Differentiation

We then tested whether inhibiting PKG could affect ES cell differentiation into cardiomyocytes. We found that 2-3 days after treatment, beating areas in DT-3-treated EBs were markedly larger than untreated controls (Figure 2(c)). The same EBs examined in Figure 2(c) were measured for total EB area (Figure 2(d)), showing that control EBs and DT-3-treated EBs were the same size, thus providing evidence that inhibiting PKG can generate more cardiomyocytes than untreated controls. It is important to note the timing of when PKG and PKC inhibitors are added. We always added inhibitors on day 3 after plating EBs into 24-well dishes. Adding inhibitors prior to this (e.g., when EBs were in suspension dishes), after day 3, or after beating had already begun did not result in significantly more beating areas when compared to controls. In fact, adding inhibitors prior to plating prevented EBs from growing as large as untreated EBs. We do not know the reason for this finding, but we speculate that, while in suspension, ES cells need active PKG and PKC for proliferation. Beating areas were measured using a reticule in the eyepiece of a dissecting microscope or after EBs were stained by immunocytochemistry. 

### 3.3. Combining PKG and PKC Isotype-Specific Inhibitors Promotes ES Cell-Derived Cardiomyocyte Differentiation

When ES cells are coaxed down the cardiomyocyte pathway, usually a small percentage of ES cells become cardiomyocytes [5, 6, 17–19]. We previously showed that molecular manipulation of the PKC pathway increased that percentage significantly [6] similar to manipulating the PKG pathway (above). Based on these observations, we surmised that if both pathways were inhibited significantly more cardiomyocytes would result when compared to manipulation of either pathway alone or controls. 

**Figure 3:**
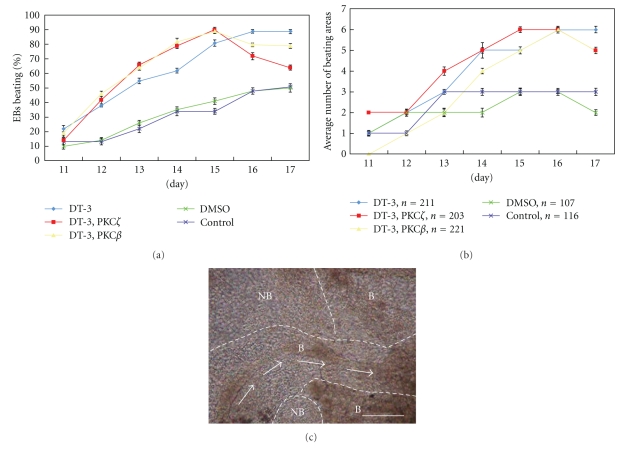
Two days after plating each EB into a well of a 24-well plate (nine days after removal of LIF), DT-3 and DT-3 + PKC*β* inhibitor, or DT-3 + PKC*ζ* inhibitor were added to different EBs. Carrier alone and untreated EBs were used as controls. EBs were then analyzed for beating two days later (Day 11 post LIF removal). As an added control, all four authors routinely performed blind counts of EBs to decrease subjectiveness. Discrepancies between authors were rare, but when they occurred, the morphology observed by the majority of authors was used as the data point. (a) Inhibiting PKG alone or in combination with PKCbeta or zeta resulted in significantly more EBs with at least one beating area when compared to controls. Significance is shown in Table 1 at each time point. The combination of DT-3 + PKCbeta or zeta inhibitors revealed in a trend towards more EBs that had at least one beating area when compared to DT-3 alone. Significance (*P* < .05) was attained on Day 14. (b) Inhibiting PKG alone or in combination with PKCbeta or zeta resulted in significantly more beating areas within EBs when compared to controls. Significance is shown in Table 2 at each time point. (c) A typical Day 14 EB outgrowth that has been subjected to DT-3 and PKCbeta specific inhibitor. NB = nonbeating area, B = beating area. Arrows depict the directional flow of contraction of the larger beating area. Scale bar = 100 *μ*meter. The number of EBs analyzed for both (a) and (b) is depicted below their specific treatment (e.g., *n* = 211 EBs for DT-3 treatment).

 Analyzing over 100 EBs for each treatment, we routinely observed significantly more EBs beating sooner after incubation in DT-3 or DT-3 + either PKC inhibitor when compared to control (Figure 3(a)). On average, by day 12 of differentiation, 10% untreated/DMSO control EBs had beating areas, while ~40% treated EBs contained beating areas. This trend continued throughout the duration of the experiment. 

The combinatorial effect of inhibiting PKG and PKC on cardiomyocyte differentiation became clear when individual beating areas were counted and averaged (Figure 3(b)). By day 12, EBs treated only with DT-3 had a 20% difference in beating areas versus controls, whereas by day 16 there was a 40% difference between DT-3-inhibited EBs versus controls. EBs treated with both DT-3 and PKC inhibitor had a substantial percentage that peaked at an average of 55% on day 15. 

Beating patterns unique to PKG- and PKC*β*-inhibited EBs were also observed. After 14 days in culture, beating areas within PKG- and PKC*β*−inhibited EBs covered approximately 2X the total area of control EBs (Figure 3(c)); however, overall EB sizes were not affected by inhibitor either alone or in combination. They also only exhibited minor cell death around the edges (analyzed by trypan blue, data not shown). Interestingly, contraction seemed to “flow” between two large, concurrent beating areas in PKG/PKC-inhibited EBs, whereas beating areas in control EBs were normally isolated.

Beating frequency was also measured; however, no significant difference was observed with respect to cardiomyocytes differentiated with or without PKC/PKG inhibitors. Control EBs beat rate was 78 + 5 beats/min, while beating frequency for EBs treated with PKC*β*, PKG, and PKC*β*/PKG inhibitors was 70 ± 6 beats/min, 68 ± 5 beats/min, and 65 ± 5 beats/min, respectively. 

### 3.4. Fluorescence-Activated Cell Sorting Reveals a Shift in EB-Derived Cardiomyocyte Differentiation

**Figure 4:**
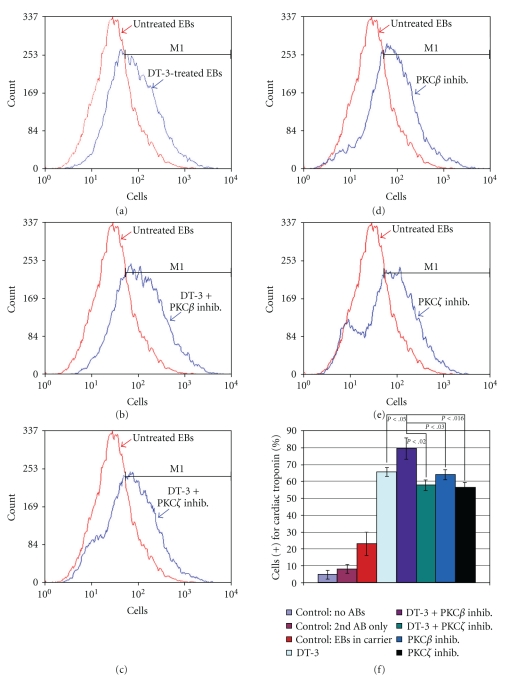
FACS analysis was used to quantify the percentage of cardiomyocytes differentiated from ES cells using a conventional protocol (control: DMSO carrier + no drugs [5, 6, 17]) and using PKG and/or PKC isotypic inhibitors. (a)–(e) Representative raw FACS data. The *x* axis is the fluorescence level of cardiac troponin. The y axis is the cell count. M1 marks cardiac troponin (+) cells. (a) Inhibiting PKG alone (blue line) markedly pushed more cells towards the cardiomyocyte lineage when compared to control EBs differentiated down the cardiomyocte pathway (red line). (b)-(c) However, combining PKG and PKC inhibitors skewed ES cell differentiation further towards the cardiomyocyte lineage when compared to DT-3-treated EBs (a) and control EBs with the highest number of cardiac troponin-positive cells found in EBs treated with DT-3 and PKCbeta specific inhibitor (b). (d)-(e) PKC inhibitors alone also increased the number of cardiomyocytes generated from ES cells but not to the extent of dual inhibition of PKCbeta and PKG. (f) Proportions of cardiac troponin expressing cells from four experiments were analyzed using the two-sample test for binomial proportions. The proportion of CCE cells expressing cardiac troponin was significantly highest in PKG/PKCbeta-(purple bar) inhibited cells when compared to EBs differentiated without drugs (red bar; *P* < .001). Inhibiting both PKC*β* and PKG resulted in the most significant increase in cardiomyocytes when compared to all other treatments (*P* values are shown for each comparison). Two types of controls were used for FACS analyses: (1) FACS machine parameters were set by analyzing cells from differentiated EBs *not* subjected to any antibodies and cells from differentiated EBs subjected to only secondary antibodies, and (2) cells from treated EBs were compared to cells from untreated (or DMSO carrier) EBs subjected to primary (anticardiac troponin) and secondary antibodies. EBs differentiated without drugs (i.e., untreated) or without drugs but in DMSO carrier demonstrated virtually identical proportions of differentiated cardiomyocytes (data not shown).

Beating areas were clearly larger in treated EBs; however, it was difficult to discern whether more cells were beating or, alternatively, the same number of beating cells as found in controls was simply dispersed over a larger portion of the EB. To determine which scenario was correct, we subjected treated and untreated EBs to FACS, which revealed that the type of treatment affected the production of ES cell-derived cardiomyocytes. Although all treatments markedly increased the number of cardiomyocytes when compared to control EBs, the highest percentage of cardiomyocytes was found in EBs treated with DT-3 + PKC*β* inhibitor (Figure 4). These results confirm that manipulation of distinct signal transduction pathways within EBs can coax more cells to become cardiomyocytes than untreated control EBs.

### 3.5. qRT-PCR Suggests That PKG Act at the Gene Level of Cardiomyocyte Differentiation

In the following experiments, we compared untreated EBs with EBs treated with DT-3 alone or DT-3 + PKC*β* at day 12 post LIF removal. We chose this time point based on our observations in Figure 3, which showed that cardiomyocytes were observed significantly sooner in DT-3 or DT-3 + PKC*β*   inhibitor-treated EBs when compared to untreated EBs. 

**Figure 5:**
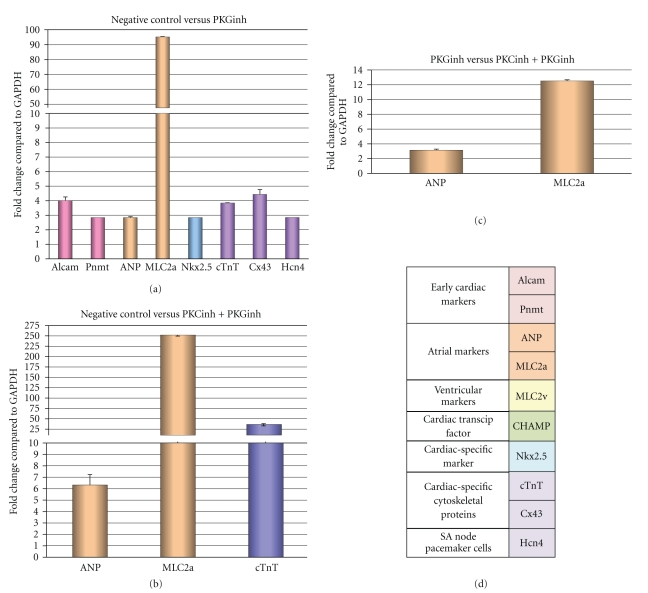
PKG in combination with PKC increases cardiac markers as shown by qRT-PCR. The time points in (a)–(c) are equivalent to Day 12 in Figures 3(a) and 3(b). (a) Negative control (untreated) versus PKG inhibitor: treatment of EBs with DT-3 resulted in increased early cardiac markers, atrial cardiac markers, the cardiac marker, Nkx2.5, as well as cardiac-specific cytoskeleton proteins when compared to negative control (untreated) EBs. (b) negative control versus combined treatment of PKC*β* inhibitor and DT-3: The combined treatment of PKC*β* and PKG inhibitors significantly upregulated atrial cardiac markers and the cardiac troponin T gene. (c) DT-3 Treatment versus combined treatment of PKC*β* inhibitor and DT-3: the combined treatment of PKC and PKG significantly upregulated both atrial markers, ANP, and MLC2a, when compared to treatment with PKG alone. (d) Table describing cardiac markers used for qRT-PCR. Graph color code = bars in graphs.

When compared to untreated EBs at the same day, at least 8 cardiac genes were upregulated in EBs treated with DT-3 + PKC*β* inhibitors (Figure 5(a)). Interestingly, specific cardiac genes were enhanced when DT-3 + PKC*β* inhibitors were combined (Figure 5(b)). In DT-3-treated EBs, ANP was increased 2.87-fold (Figure 5(a)), while ANP was increased 6.37-fold in DT-3 + PKC*β* inhibitor-treated cells (Figure 5(b)). MLC2a was increased 95.24-fold in DT-3-treated cells (Figure 5(a)); however, it was increased 251.29-fold in DT-3 + PKC*β* inhibitor-treated cells (Figure 5(b)). DT-3 + PKC*β* inhibitor-treated cells also had higher expression of the cardiac-specific cytoskeleton marker, cTnT (37.50-fold), when compared to DT-3-treated cells (3.85-fold). 

Interestingly, when comparing EBs treated with DT-3 alone versus DT-3 + PKC*β* inhibitors at day 12 post LIF removal, we found that atrial markers were upregulated in DT-3 + PKC*β* inhibitor-treated EBs. When all data are combined, they support our hypothesis that simultaneous inhibition of PKC and PKG increases the number of cardiomyocytes when compared to untreated EBs, and in addition, downregulation of PKC*β* and PKG appears to guide them down the atrial pathway.

## 4. Discussion

Signal transduction mechanisms involving PKG1 have been studied in many systems; however, the role of PKG1 in initial cardiomyocyte differentiation is not well understood [20, 21]. A previous study showed that PKG1 protein and gene expression are reduced in beating cardiomyocytes [22]. Here we show that physically down regulating PKG in ES cells during cardiomyogenesis generates more cardiomyocytes than untreated controls. These results support our hypotheses that downregulating PKG1 stimulates ES cell differentiation into cardiomyocytes and that combining PKG/PKC specific inhibitors results in significantly more cardiomyocytes when compared to either inhibitor alone or untreated controls. 

Why would PKG and PKC*β* and *ζ* need to be downregulated in beating areas? One answer is that PKG can be activated by nitric oxide (NO) through activation of guanylate cyclase, which converts GTP into cGMP [7–9]. NO is continually present in ES cells especially during stages of differentiation prior to the cardiac lineage [22, 23]. With the evidence from past investigations demonstrating that cGMP attenuates muscle contraction in adult cardiomyocytes [11, 24] by inhibiting the cardiac L-type CA^2+^ channel current (*I*_Ca,L_) and, along with PKA, the voltage-dependent fast Na^+^ current in ventricular myocytes [11], we conclude that ES cells must downregulate PKG to enable activation of the cardiomyocyte electrical system. Also, in other systems, PKG can phosphorylate MLC phosphatase resulting in inhibition of contraction [10, 24], which could further impair contraction in ES cell-derived cardiomyocytes if it is not downregulated. The purpose for downregulating PKC has been described in [6]. 

However, another answer is that PKG and PKC could have been recruiting more ES cells to differentiate into cardiomyocytes. DT-3 has been shown to only affect the activity of PKG and not to affect gene transcription [25–28]. As a result we propose that the upregulation of specific cardiac genes is due to a release of a putative PKG/PKC*β* repression mechanism. Once PKG and/or PKC*β* are downregulated, more cells within an EB can be recruited to enter the cardiac pathway as PKG and PKC cardiac repressors involved in transcription can become deactivated. PKG has been shown to regulate gene expression under certain circumstances [29] as has PKC [30].

Substantial research promise includes applying these signal transduction findings to human adult stem cells derived from cord blood, bone marrow, or the new iPS. Though it is currently believed that adult stem cells are not as flexible as ES cells, it might be possible to apply these methods to transdifferentiate significantly more adult stem cells into embryonic-like cardiomyocytes for therapeutic use. We are currently investigating the possibility of applying these same PKG and PKC inhibitors to mesenchymal stem cells. The results of this study could contribute to the efforts aimed at the ultimate goal of circumventing the harmful affects of natural cardiac remodeling and thus safely rebuilding hearts damaged by heart attack or congestive heart failure.

## Figures and Tables

**Table 1:** Significance comparing treated groups to controls (for data points in Figure 3(a)) % of EBs beating.

DT-3 compared to controls					
Day 12: *P* < .02	Day 13: *P* < .002	Day 14: *P* < .008	Day 15: *P* < .005	Day 16: *P* < .002	Day 17: *P* < .002

DT-3 + PKC*ζ* inhibitor compared to controls					

Day 12: *P* < .04	Day 13: *P* < .0005	Day 14: *P* < .001	Day 15: *P* < .0001	Day 16: *P* < .001	Day 17: *P* < .05

DT-3 + PKC*β* inhibitor compared to controls					

Day 12: *P* < .001	Day 13: *P* < .003	Day 14: *P* < .0008	Day 15: *P* < .001	Day 16: *P* < .001	Day 17: *P* < .003

**Table 2:** Significance comparing treated groups to controls (for data points in Figure 3(b)) number beating areas. NS: no significance.

DT-3 compared to controls					
Day 12: NS	Day 13: NS	Day 14: NS	Day 15: NS	Day 16: *P* < .006	Day 17: *P* < .001

DT-3 + PKC*ζ* inhibitor compared to controls					

Day 12: NS	Day 13: *P* < .0001	Day 14: *P* < .0001	Day 15: *P* < .0006	Day 16: *P* < .006	Day 17: *P* < .02

DT-3 + PKC*β* inhibitor compared to controls					

Day 12: NS	Day 13: NS	Day 14: *P* < .02	Day 15: *P* < .02	Day 16: *P* < .001	Day 17: *P* < .02
